# No association between *APOE* genotype and lipid lowering with cognitive function in a randomized controlled trial of evolocumab

**DOI:** 10.1371/journal.pone.0266615

**Published:** 2022-04-11

**Authors:** Laura E. Korthauer, Robert P. Giugliano, Jianping Guo, Marc S. Sabatine, Peter Sever, Anthony Keech, Dan Atar, Christopher Kurtz, Christian T. Ruff, Francois Mach, Brian R. Ott

**Affiliations:** 1 Alpert Medical School of Brown University, Providence, RI, United States of America; 2 Rhode Island Hospital, Providence, RI, United States of America; 3 TIMI Study Group, Brigham and Women’s Hospital, Harvard Medical School, Boston, MA, United States of America; 4 Imperial College London, London, United Kingdom; 5 University of Sydney, Sydney, NSW, Australia; 6 Oslo University Hospital Ulleval, Department of Cardiology and University of Oslo, Oslo, Norway; 7 Amgen, Newbury Park, CA, United States of America; 8 Geneva University Hospitals, Geneva, Switzerland; Università degli Studi di Milano, ITALY

## Abstract

*APOE* encodes a cholesterol transporter, and the ε4 allele is associated with higher circulating cholesterol levels, ß-amyloid burden, and risk of Alzheimer’s disease. Prior studies demonstrated no significant differences in objective or subjective cognitive function for patients receiving the PCSK9 inhibitor evolocumab vs. placebo added to statin therapy. There is some evidence that cholesterol-lowering medications may confer greater cognitive benefits in *APOE* ε4 carriers. Thus, the purpose of this study was to determine whether *APOE* genotype moderates the relationships between evolocumab use and cognitive function. *APOE*-genotyped patients (N = 13,481; 28% ε4 carriers) from FOURIER, a randomized, placebo-controlled trial of evolocumab added to statin therapy in patients with stable atherosclerotic cardiovascular disease followed for a median of 2.2 years, completed the Everyday Cognition Scale (ECog) to self-report cognitive changes from the end of the trial compared to its beginning; a subset (N = 835) underwent objective cognitive testing using the Cambridge Neuropsychological Test Automated Battery as part of the EBBINGHAUS trial. There was a dose-dependent relationship between APOE ε4 genotype and patient-reported memory decline on the ECog in the placebo arm (*p* = .003 for trend across genotypes; ε4/ε4 carriers vs. non-carriers: OR = 1.46, 95% CI [1.03, 2.08]) but not in the evolocumab arm (*p* = .50, OR = 1.18, 95% CI [.83,1.66]). However, the genotype by treatment interaction was not significant (*p* = .30). In the subset of participants who underwent objective cognitive testing with the CANTAB, APOE genotype did not significantly modify the relationship between treatment arm and CANTAB performance after adjustment for demographic and medical covariates, (*p*’s>.05). Although analyses were limited by the low population frequency of the ε4/ε4 genotype, this supports the cognitive safety of evolocumab among ε4 carriers, guiding future research on possible benefits of cholesterol-lowering medications in people at genetic risk for Alzheimer’s disease.

## Introduction

Use of the proprotein convertase subtilisin-kexin type 9 (PCSK9) inhibitor evolocumab reduces low-density lipoprotein (LDL) cholesterol by ~60% and lowers the rate of cardiovascular events among patients with established cardiovascular disease [1]. PCSK9 is primarily expressed in the liver, where it binds the low-density lipoprotein receptor on the surface of hepatocytes [2]. It is also expressed in the brain, where it modulates cortical neuronal differentiation and apoptosis [3].

While cardiovascular disease is a risk factor for cognitive decline and Alzheimer’s disease [4–6], there has been debate regarding the effects of statins on cognition. Two studies along with case reports have suggested mild adverse effects [7–9], while other studies, including several meta-analyses, suggest neutral or even positive effects [10–12]. Assessment of the influence of genetic makeup and other factors may lead to a better understanding of the cognitive effects of lipid-lowering medications.

Apolipoprotein E (*APOE*) is a gene that encodes a cholesterol transporter, and the ε4 allele is associated with higher circulating cholesterol levels [13], greater burden of cortical ß-amyloid plaques [14–16], and elevated risk for Alzheimer’s disease (AD) [17, 18]. As early as middle age, cognitively normal *APOE* ε4 carriers report greater subjective cognitive complaints [19, 20] and perform more poorly than non-ε4 carriers on tests of memory and executive functioning [21–25]. The conjunction of *APOE* ε4 genotype and hypercholesterolemia has been associated with even greater cognitive decline than either risk factor alone [26, 27]. This suggests that cholesterol-lowering medications may confer greater cognitive benefits for *APOE* ε4 carriers than non-ε4 carriers.

A recent re-analysis of patient-level data from multiple AD clinical trials found that long-term use of various statins resulted in lower rates of cognitive decline, with potentially greater therapeutic efficacy in *APOE* ε4/ε4 carriers [28]. These trials included patients with mild cognitive impairment (MCI) or dementia due to AD.

The present study seeks to determine whether this pattern of benefit extends to cognitively normal adults. We focus on cognitively normal patients enrolled in FOURIER, a double-blind randomized placebo-controlled trial of evolocumab added to statin therapy. The FOURIER trial showed that combination evolocumab and statin therapy reduced the risk of major cardiovascular events by 15% and had no significant adverse effect on self-reported cognition or neurocognitive adverse events [1, 29]. A subgroup of 1,204 patients participated in EBBINGHAUS (Evaluating PCSK9 Binding Antibody Influence on Cognitive Health in High Cardiovascular Risk Subjects), a study to investigate objective neurocognitive functioning using the Cambridge Neuropsychological Test Automated Battery (CANTAB). Results from EBBINGHAUS showed no differences in cognitive function between patients receiving evolocumab or placebo in addition to statin therapy [30].

While these primary analyses established the cognitive safety of evolocumab, its potential cognitive benefits for subgroups of patients based on *APOE* genotype is unknown. We investigated *APOE* genotype as a moderator of the relationship between evolocumab and patient-reported cognitive impairment (assessed in the larger FOURIER cohort) and objective cognitive performance (assessed in the EBBINGHAUS subgroup). We hypothesized that *APOE* ε4 carriers would show a greater benefit of cholesterol-lowering medications than non-ε4 carriers. Specifically, we predicted that *APOE* ε4 carriers would show cognitive decline over the course of the study and that this decline would be less in those receiving active treatment with evolocumab.

## Methods

### Study population and randomization

Participants in the FOURIER trial [31] (ClinicalTrials.gov identification number NCT01764633; protocol DOI: 10.1016/j.ahj.2015.11.015) were aged 40 to 85 years; had clinically evident atherosclerotic cardiovascular disease, defined as a history of myocardial infarction, symptomatic peripheral artery disease, or nonhemorrhagic stroke; fasting LDL cholesterol level of 70 mg/dL or higher or a non-high-density lipoprotein (HDL) cholesterol level of 100 mg/dL or higher while taking an optimized regimen of moderate- or high-intensity statin therapy. LDL-C was estimated using the Friedewald equation unless the LDL-C was <40 mg/dL or the triglycerides were ≥400 mg/dL, in which case the LDL-C was directly measured using preparative ultracentrifugation. Patients with a current or past diagnosis of mild cognitive impairment or dementia were excluded from participation. Eligible patients at centers from 49 countries were assigned in a 1:1 ratio to receive subcutaneous injections of evolocumab (either 140 mg every 2 weeks or 420 mg every month, according to patient preference) or matching placebo. For the subset of participants in the EBBINGHAUS study (ClinicalTrials.gov trial identification number NCT02207634), enrollment was encouraged to occur before the administration of the first dose of the study drug or placebo in the FOURIER trial, although enrollment was permitted until the 12-week visit. All patients provided written informed consent. The protocols for FOURIER and EBBINGHAUS were approved by ethics committees at each participating center.

### Genotyping

A subset of participants consented to provide a blood sample for *APOE* genotyping. Samples were genotyped on the Infinium Global Screening Array chip. *APOE* genotypes were defined by the two common single nucleotide polymorphisms (SNPs) of the *APOE* gene: 388 T>C (rs429358) and 526 C>T (rs7412).

### Endpoints

For the FOURIER trial, patient-reported (subjective) cognition was measured at the final study visit. Patients completed an abbreviated 23-item questionnaire that included the executive functioning and memory subscales of the Everyday Cognition (ECog) scale [32]. Participants were asked to rate their current level of cognitive performance in comparison to the beginning of the study. Change over time was rated on a four-point scale, with lower scores representing better cognitive functioning: 1) better or no change; 2) questionable or occasionally worse; 3) consistently a little worse; 4) consistently much worse. The memory domain includes questions about recalling the content of conversations, location of everyday objects, and important dates or appointments. The executive functioning domain evaluates abilities in three subdomains: planning, organization, and divided attention. Given the ordinal scale used in this measure, ECog scores were compared as a binary outcome (1, indicating no change, versus >1, indicating self-reported cognitive change).

Objective cognitive performance was assessed in the EBBINGHAUS trial using CANTAB (www.cambridgecognition.com), a computerized battery of language-independent neuropsychological tests that is sensitive to detect cognitive dysfunction related to cerebrovascular disease [33] and Alzheimer’s disease [34]. The CANTAB assessments were performed at the screening visit (training session), baseline, 24 weeks, annually, and at the end of the trial. Three CANTAB subtests were used: 1) Spatial Working Memory, in which participants find a target by systematically searching an array of boxes (outcomes include errors and a strategy index score); 2) Paired Associates Learning, a visuospatial associative learning task (outcome is total errors adjusted for the estimated number of errors on stages not reached; 3) Reaction Time (RT), in which participants release a button upon the onset of a colored stimulus in a 5-circle array (outcome is median RT). For each of the CANTAB outcomes, a z-score was calculated for each participant, comparing that individual’s score to the mean baseline scores for all participants. A global composite score was calculated by averaging the combined z-scores of each endpoint; higher scores indicate better performance.

### Statistical analysis

Baseline characteristics between *APOE* carrier and *APOE* non-carriers were compared using Wilcoxon rank-sum tests for continuous variables and the χ-^2^ test for categorical variables.

ECog scores were compared as a binary outcome (1, indicating no change, versus >1, indicating self-reported cognitive change) among subjects with a different number of copies of the *APOE* ε4 allele using logistic regression models. Following are the variables used in the adjusted logistic regression models: randomized stratification factors (randomized allocation of study treatment was based on final screening LDL-C [<85 mg/dL vs > = 85 mg/dL] and geographic location), age, sex, race, prior stroke, high/moderate intensity statin, CHA_2_DS_2_-VASc Score> = 4, current smoker, prior atrial fibrillation, cerebrovascular disease, hypertension, diabetes and non-stroke neurologic disease. Trend across genotypes (0, 1, or 2 ε4 alleles) and a genotype x treatment interaction were also assessed.

For longitudinal CANTAB scores, repeated measure mixed-effects models were utilized to estimate treatment difference (by comparing least square means between the placebo and evolocumab groups averaged over time) and to examine whether *APOE* genotype moderates the association between evolocumab use and cognitive function through an interaction term (*APOE* ε4 carrier status by treatment group). These mixed models were further adjusted by baseline CANTAB score (standardized), level of education, and the additional covariates listed above for ECog analyses.

Comparisons of CANTAB scores between treatment groups used repeated measures mixed-effect linear models within each *APOE* ε4 subgroups. Adjusted models used the same covariates as the ECog analyses as well as baseline CANTAB score (z-score), visit number, and education.

Statistical significance was assessed at a nominal α level of 0.05. All reported P values were 2 sided. All statistical computations were performed with SAS System V9.4 (SAS Institute Inc, Cary, NC).

## Results

### Patients

**Fig 1:**
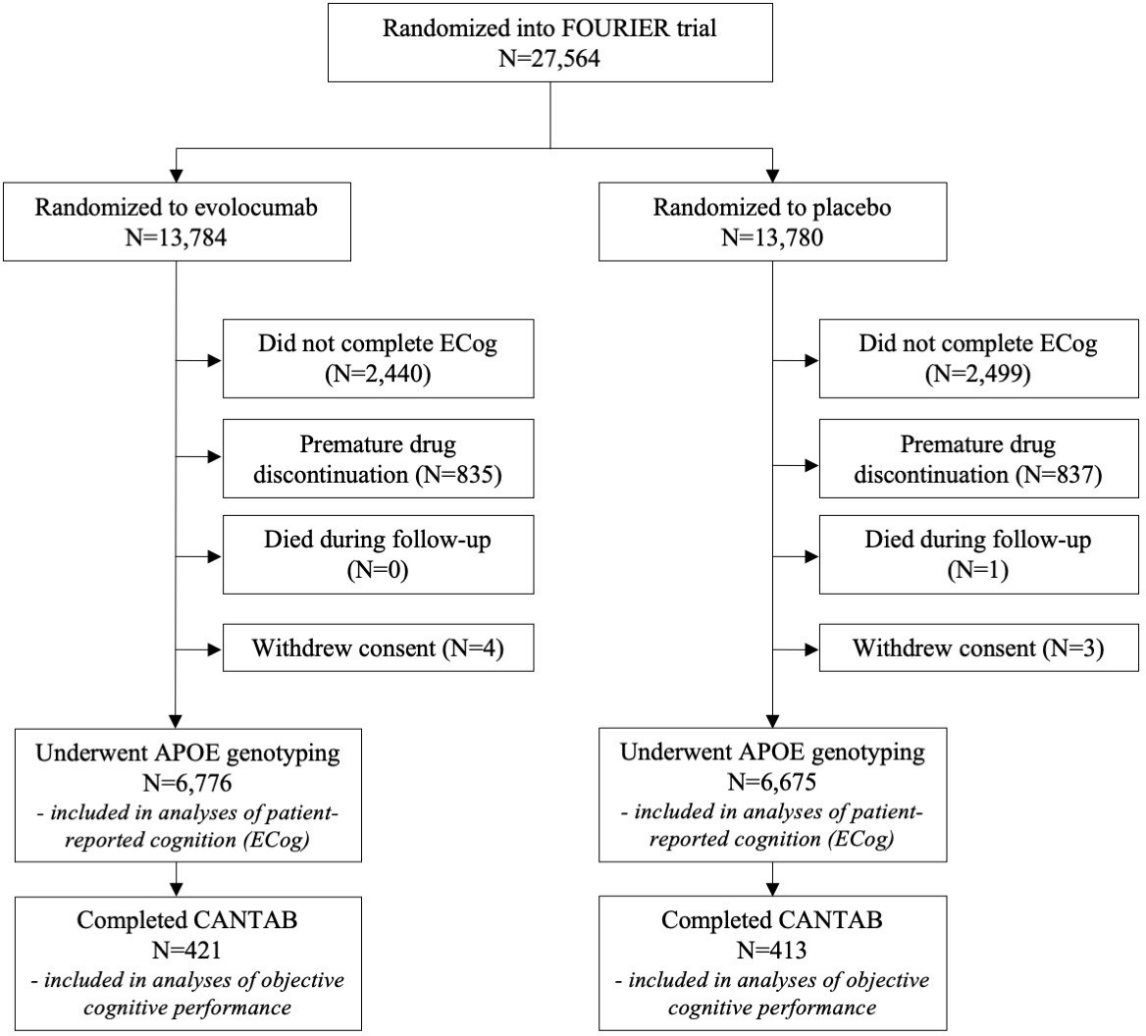
CONSORT diagram.

From February 2013 through June 2015, a total of 27,564 patients enrolled in the FOURIER trial and were randomized (see CONSORT diagram, Fig 1). Of these, 13,481 underwent *APOE* genotyping and completed the ECog, while 834 participants with *APOE* genotyping completed the CANTAB assessments. Of those who underwent *APOE* genotyping, 36 (0.3%) were ε2/ε2, 816 (6.1%) were ε2/ε3, 190 (1.4%) were ε2/ε4, 8,827 (65.6%) were ε3/ε3, 3,279 (24.4%) were ε3/ε4, and 303 (2.3%) were ε4/ε4 carriers. Allele frequencies were in Hardy-Weinberg equilibrium. Baseline demographic characteristics of ε4 carriers were broadly similar to non-ε4 carriers, though ε4 carriers were younger, more likely to have prior myocardial infarction, and less likely to have had a prior stroke, CHADS-VASc score for atrial fibrillation ≥ 4, diabetes mellitus, or report current cigarette use. (Table 1). The ε4 carriers had significantly higher LDL-C at baseline than non-ε4 carriers. The mean duration of follow-up was 2.3 years (*SD* = .42; range = 1.37 to 3.60).

**Table 1:** Baseline characteristics.

Characteristics	*APOE* non-ε4 (N = 9679)	*APOE* ε2/ε4 or ε3/ε4 (N = 3469)	*APOE* ε4/ε4 (N = 303)	P Value
Age, years (median,IQR)	62.63 (8.9)	62.14 (8.8)	61.30 (8.0)	0.002
Male, n(%)	7401 (76.5%)	2646 (76.3%)	219 (72.3%)	0.24
Caucasian, n(%)	8888 (91.8%)	3156 (91.0%)	275 (90.8%)	0.26
Region				<0.001
North America, n(%)	1271 (13.1%)	541 (15.6%)	51 (16.8%)	
Europe, n(%)	7306 (75.5%)	2513 (72.4%)	220 (72.6%)	
Latin America, n(%)	121 (1.3%)	35 (1.0%)	1 (0.3%)	
Asia Pacific, n(%)	981 (10.1%)	380 (11.0%)	31 (10.2%)	
Prior myocardial infarction, n(%)	7805 (80.6%)	2879 (83.0%)	260 (85.8%)	0.001
Prior stroke, n(%)	1753 (18.1%)	582 (16.8%)	41 (13.5%)	0.034
Peripheral arterial disease, n(%)	1362 (14.1%)	450 (13.0%)	49 (16.2%)	0.135
CHADS-Vasc > = 4, n(%)	4418 (45.7%)	1485 (42.8%)	123 (40.6%)	0.005
Non-stroke neurological findings, n(%)	902 (9.3%)	332 (9.6%)	35 (11.6%)	0.40
Atrial fibrillation (at any time), n(%)	844 (8.7%)	266 (7.7%)	16 (5.3%)	0.02
Congestive heart failure, n(%)	1951 (20.2%)	674 (19.4%)	55 (18.2%)	0.48
Hypertension, n(%)	7703 (79.6%)	2684 (77.4%)	237 (78.2%)	0.024
Diabetes mellitus, n(%)	3422 (35.4%)	1156 (33.3%)	84 (27.7%)	0.004
Current cigarette use, n(%)	2768 (28.6%)	913 (26.3%)	77 (25.4%)	0.02
Statin Use				
High intensity, n(%)	6773 (70.0%)	2491 (71.8%)	236 (77.9%)	0.003
Moderate intensity, n(%)	2875 (29.7%)	971 (28.0%)	67 (22.1%)	0.004
Ezetimibe, n(%)	552 (5.7%)	238 (6.9%)	28 (9.2%)	0.003
Aspirin or P2Y12 inhibitor, n(%)	8936 (92.3%)	3205 (92.4%)	289 (95.4%)	0.14
Betablocker, n(%)	7481 (77.3%)	2661 (76.7%)	240 (79.2%)	0.55
ACE inhibitor or ARB or aldosterone antagonist, n(%)	7772 (80.3%)	2751 (79.3%)	238 (78.6%)	0.37
LDL-C, mg/dL (median, IQR)	97.30 (27.5)	98.41 (27.3)	103.21 (28.5)	<0.001
Total cholesterol, mg/dL (median IQR)	174.12 (32.7)	174/19 (32.5)	178.20 (35.2)	0.04
HDL-C, mg/dL (median,IQR)	46.88 (12.7)	45.73 (12.6)	46.46 (14.2)	<0.001
Triglycerides, mg/dL (median,IQR)	150.46 (69.5)	151.05 (71.6)	145.58 (66.2)	0.45
Lipoprotein(a), nmol/L (median IQR)	90.92 (109.6)	94.01 (117.1)	99.18 (123.4)	0.85
ECog Memory ≥ 1, n(%)	2859 (29.5%)	1064 (30.7%)	103 (34.0%)	0.14
ECog Executive Functioning ≥ 1,n(%)	2491 (25.8%)	857 (24.7%)	84 (27.7%)	0.32
Total score ≥ 1,n(%)	3324 (34.3%)	1229 (35.4%)	116 (38.3%)	0.22
Follow-up time, yrs(median,IQR)	2.32 (0.4)	2.34 (0.4)	2.37 (0.4)	0.10

*Note*: This sample reflects the 13,451 participants who had both ECog data and *APOE* genotyping.

### Patient-reported cognition: ECog

In light of our *a priori* hypotheses, we started by examining the main effects of *APOE* genotype on ECog scores for each treatment arm. In the placebo arm, there was a dose-dependent relationship between *APOE* ε4 genotype and patient-reported memory decline (*p* = .003 for trend across genotypes; ε4/ε4 carriers vs. non-carriers: OR = 1.46, 95% CI [1.03, 2.08]) and total ECog score (*p* = .009 for trend across genotypes; OR = 1.33, 95% CI [.94, 1.89]), but not executive functioning subscore (*p* = .77 for trend across genotypes; OR = 1.15, 95% CI [.78, 1.69]). In the evolocumab arm, there was no significant relationship between *APOE* genotype and memory (*p* = .50 for trend across genotypes), executive functioning (*p* = .31 for trend across genotypes), or total ECog score (*p* = .39 for trend across genotypes. After adjusting for relevant covariates, there were no significant interactions between *APOE* genotype and treatment arm for total ECoG score (*p* = .92) or the memory (*p* = .30) or executive functioning (*p* = .74) subscales (Table 2).

**Table 2:** ECog scores by treatment arm and *APOE* ε4 allele (adjusted models).

		non-ε4	1 ε4 allele	ε4/ε4	1 ε4 allele vs. non-ε4		ε4/ε4 vs. non-ε4		Trend across genotypes (0, 1, or 2 ε4 alleles)	
ECog Domain	Treatment	n (%)	n (%)	n (%)	Odds Ratios1 (95% CI)	P-value	Odds Ratios2 (95% CI)	P-value	P-value	P-interaction
Memory >1	Placebo	1399 (29.3%)	552 (31.7%)	52 (35.9%)	1.16(1.03,1.31)	0.0179	1.46(1.03,2.08)	0.035	0.003	0.30
	Evolocumab	1460 (29.8%)	512 (29.6%)	51 (32.3%)	1.01(0.90,1.15)	0.8145	1.18(0.83,1.66)	0.35	0.50	
Executive Functioning >1	Placebo	1206 (25.2%)	423 (24.3%)	37 (25.5%)	1.00(0.87,1.13)	0.9531	1.15(0.78,1.69)	0.49	0.77	0.74
	Evolocumab	1285 (26.3%)	434 (25.1%)	47 (29.8%)	0.96(0.85,1.10)	0.5805	1.20(0.84,1.71)	0.31	0.93	
Total Score >1	Placebo	1602 (33.5%)	622 (35.8%)	55 (37.9%)	1.14(1.02.1.29)	0.0260	1.33(0.94,1.89)	0.11	0.009	0.92
	Evolocumab	1722 (35.2%)	607 (35.1%)	61 (38.6%)	1.02(0.91.1.15)	0.7168	1.20(0.86,1.67)	0.28	0.40	

*Note*. Adjusted logistic regression models used the following covariates: stratification factors, age, sex, race, prior stroke, high/moderate intensity statin, CHA2DS2-VASc Score> = 4, current smoker, prior atrial fibrillation, cerebrovascular disease, hypertension, diabetes and non-stroke neurologic disease.

### Objective cognition: CANTAB

In the 835 participants who completed objective cognitive testing with the CANTAB, there were no main effects of *APOE* genotype on CANTAB scores for either treatment arm (Table 3). There were also no significant interactions between *APOE* genotype and treatment arm after adjusting for relevant covariates (Table 3).

**Table 3:** CANTAB scores by treatment arm and *APOE* ε4 allele (adjusted models).

CANTAB Domain	Between-group Difference (Placebo vs. Evolocumab); LSmeans (95% CI)	P Value	P-Value for Interaction
Spatial Working Memory Strategy Index			
non-ε4	0.017 (-0.080,0.114)	0.73	0.16
ε4	0.172 (-0.002,0.346)	0.05	
Spatial Working Memory Between-Errors			
non-ε4	0.019 (-0.080,0.117)	0.71	0.34
ε4	0.100 (-0.061,0.262)	0.22	
Paired Associates Learning			
non-ε4	0.053 (-0.040,0.146)	0.27	0.69
ε4	0.004 (-0.145,0.154)	0.95	
Reaction Time			
non-ε4	0.080 (-0.030,0.188)	0.15	0.08
ε4	-0.090 (-0.241,0.062)	0.24	
CANTAB Global Composite Score			
non-ε4	0.038 (-0.020,0.096)	0.20	0.74
ε4	0.053 (-0.039,0.146)	0.26	

*Note*. Adjusted linear mixed effects models included the following covariates: baseline CANTAB score (z-score), visit number, education, stratification factors (from IVRS), age, sex, race, prior stroke, high/moderate intensity statin, CHA2DS2-VASc Score> = 4, current smoker, prior atrial fibrillation, cerebrovascular disease, hypertension, diabetes and non-stroke neurologic disease.

## Discussion

In this analysis of 16,174 patients with cardiovascular disease, *APOE* genotype did not significantly moderate the relationship between evolocumab treatment and patient-reported or objectively assessed decline in cognition. To our knowledge, this is the first study to investigate the potential effect of *APOE* genotype on cognition in a large randomized clinical trial of a PCKS9 inhibitor with measures of both patient-reported and objective cognitive functioning.

Prior reports [29, 30, 35] demonstrated the overall cognitive safety of evolocumab combined with statin therapy for objective and patient-reported cognition, even among patients who achieved a very low LDL-C concentration of <20 mg/dL.

In addition to evidence that dyslipidemia increases risk of Alzheimer’s disease, there are several recent reports demonstrating a link between PCSK9 and Alzheimer’s disease pathogenesis [3, 36, 37]. In rodents fed a high-fat diet, hippocampal neuronal apoptosis is associated with an increase in PCSK9 expression [38], while the PCSK9 inhibitor alirocumab reduces neuroinflammation and ameliorates cognitive deficits [39]. Furthermore, patients with Alzheimer’s disease have higher PCSK9 mRNA and protein levels in their postmortem brains [40] as well as higher cerebrospinal fluid levels of PCSK9 [41]. Given the link between *APOE* and lipid homeostasis, this led to our hypothesis that the PCSK9 inhibitor evolocumab may confer a cognitive benefit in *APOE* ε4 carriers.

The present findings support the cognitive safety of combination evolocumab and statin therapy among cognitively normal patients at higher genetic risk for Alzheimer’s disease. Though the *APOE* genotype by treatment arm interactions did not achieve statistical significance, interestingly there was a dose-dependent relationship between *APOE* genotype and patient-reported memory decline in the placebo arm but not the evolocumab arm. This provides preliminary evidence to support our hypothesis that ε4 carriers, particularly ε4/ε4 carriers who are at highest risk for Alzheimer’s disease, may show less cognitive impairment on cholesterol-lowering medications. One possible explanation for our non-significant genotype by treatment interactions is that the low population frequency of the *APOE* ε4/ε4 genotype (2.2% of the present sample) may have limited statistical power to detect effect modification, though it is also possible that no such effect is present. Nevertheless, the qualitative pattern of findings suggesting a possible cognitive benefit of evolocumab in *APOE* ε4 carriers could be used to guide future research.

There are several limitations to this study. First, patient-reported cognition on the ECog was only collected at a single timepoint at the end of the study. Thus, patients who dropped out of the study due to major adverse events were not represented in the sample. Although the drop-out rate did not differ between treatment arms (3.9%), this may have biased ECog scores toward patients with less severe cognitive complaints. Second, only a subgroup of the main trial participated in the CANTAB substudy, which objectively measured cognitive functioning. This limited our statistical power to detect an effect of evolocumab on objective cognition as well as our ability to conduct subgroup analyses in carriers of one versus two ε4 alleles. Third, the follow-up interval was relatively short (*M* = 2.3 years) to detect a change in cognitive performance. Finally, the ECog and CANTAB are not comprehensive measures of patient-reported and objective cognitive impairment. While they are well-validated and appropriate to the patient population [42, 43], they do not measure all aspects of cognition and might be less sensitive to some changes than a more comprehensive neuropsychological battery. As a self-report measure, the ECog is also susceptible to recall bias.

In summary, we report that *APOE* genotype did not significantly moderate the relationship between combination evolocumab and statin therapy and patient-reported or objective cognition in this large, randomized controlled trial. Given the small number of ε4/ε4 carriers in this sample, future research is needed to evaluate the effectiveness of PCSK9 inhibitors to reduce the risk of cognitive decline in those most at risk for Alzheimer’s disease.

## Supporting information

S1 Table ECog scores by treatment arm and *APOE* ε4 allele (unadjusted models). (DOCX)

S1 Checklist CONSORT 2010 checklist of information to include when reporting a randomised trial*. (DOC)
