# Supplementary material for: No association between APOE genotype and lipid lowering with cognitive function in a randomized controlled trial of evolocumab
Source: PLoS One. 2022 Apr 11;17(4):e0266615. doi: 10.1371/journal.pone.0266615 (PMC9000128; doi:10.1371/journal.pone.0266615)
Supplement: S1 Table — (DOCX) [file pone.0266615.s001.docx]

**Supplemental Materials**

**Supplemental Table 1. ECog scores by treatment arm and *APOE* ε4 allele (unadjusted models)**

|  |  | non-ε4 | 1 ε4 allele | ε4/ε4 | 1 ε4 allele vs. non-ε4 | | ε4/ε4 vs. non-ε4 | | Trend across genotypes (0, 1, or 2 ε4 alleles) | |
| --- | --- | --- | --- | --- | --- | --- | --- | --- | --- | --- |
| ECog Domain | Treatment | n (%) | n (%) | n (%) | Odds Ratios1 (95% CI) | P-value | Odds Ratios2 (95% CI) | P-value | P-value | P-interaction |
| Memory >1 | Placebo | 1399 (29.3 %) | 552 (31.7%) | 52 (35.9%) | 1.16(1.03.1.31) | 0.039 | 1.38(0.98,1.95) | 0.068 | 0.009 | 0.32 |
|  | Evolocumab | 1460 (29.8 %) | 512 (29.6%) | 51 (32.3%) | 1.01(0.90.1.15) | 0.91 | 1.14(0.81,1.60) | 0.46 | 0.77 |  |
| Executive Functioning >1 | Placebo | 1206 (25.2%) | 423 (24.3%) | 37 (25.5%) | 1.00(0.87.1.13) | 0.59 | 1.04(0.71,1.52) | 0.83 | 0.74 | 0.46 |
|  | Evolocumab | 1285 (26.3 %) | 434 (25.1%) | 47 (29.8%) | 0.96(0.85.1.10) | 0.35 | 1.18(0.83.1.68) | 0.35 | 0.80 |  |
| Total Score >1 | Placebo | 1602 (33.5 %) | 622 (35.8%) | 55 (37.9%) | 1.14(1.02.1.29) | 0.062 | 1.24(0.88,1.74) | 0.22 | 0.03 | 0.21 |
|  | Evolocumab | 1722 (35.2%) | 607 (35.1%) | 61 (38.6%) | 1.02(0.91.1.15) | 1.00 | 1.17(0.84,1.62) | 0.35 | 0.63 |  |
